# Classification of Human Failure in Chemical Plants: Case Study of Various Types of Chemical Accidents in South Korea from 2010 to 2017

**DOI:** 10.3390/ijerph182111216

**Published:** 2021-10-26

**Authors:** Seungho Jung, Seungkyoo Pak, Kwanwoo Lee, Chankyu Kang

**Affiliations:** 1 Department of Environmental and Safety Engineering, Ajou University, Worldcupro 206, Yeongtong-gu, Suwon 16499, Korea; processsafety@ajou.ac.kr; 2 Safety Department, Coupang, 570 Tower 730, Songpa-daero, Songpa-gu, Seoul 05510, Korea; koshachem@naver.com; 3 School of Social Safety System Engineering, Hankyong National University, Jungang-ro 327, Anseong-si 17579, Korea; dlrhksdn1102@hknu.ac.kr

**Keywords:** chemical accident, human failure, classification, HSE, RCA, MSDS

## Abstract

The increasing use and distribution of chemicals are causing serious chemical accidents such as fires, explosions, and leaks during manufacturing and handling. In most cases, all risks caused by chemicals are classified as accidents due to defects in process facilities, human errors, and multi-cause accidents. Among chemical accidents caused by human errors, accurate analysis of accidents caused by the complex action of various types of human failures is required. Based on the accident investigation reports that occurred in South Korea from 2010 to 2017, chemical accidents caused by human failure were analyzed, and the fundamental causes were derived by classifying them into human error and violation. Human failure was analyzed according to the classification criteria for human failure of health and safety executive (HSE). As a result of the analysis, several types of human failure acted in combination to be a more significant cause of chemical accidents; incorrect application of process rules and procedures, inappropriate chemical information, lack of education, and defects in the current safety regulation were analyzed as the main causes. In addition, the cause of human errors was presented through case studies of chemical accidents in South Korea.

## 1. Introduction

The complexity and the automation of a chemical plant industry (CPI) have impacted human life profoundly by producing many essential products, utilizing a variety of new chemicals. In many aspects, the increased application of chemical products has not only improved the quality of human life but also increased dependence of humans on these chemicals [1]. However, chemicals pose potential threats to human life and the environment because of their flammability, reactivity, and toxicity [2,3]. These properties can cause fire outbreak and explosion and exert deleterious effects on human health [4]. Disastrous chemical accidents, including Flixborough’s UVCE explosion (28 deaths in 1974), Seveso’s dioxin emissions (2000 population exposures in 1976), methyl isocyanate (MIC) leakage in Bhopal (350 deaths in 1984), storage tank explosion in Buncefield (2005), petrochemical plant explosions in Mexico (37 deaths, 2013), hydrofluoric acid leak in South Korea (5 deaths in 2012), and explosion in Tenjin (50 deaths in 2015), have resulted in deaths as well as property damages, as already stated [5,6,7,8,9].

For a systematic approach toward preventing these chemical accidents, chemical manufacturers must convey the right information about the potential hazards and the correct safety precautions to users [10]. The International Labor Organization (ILO) code of practice for safety in the handling and the utilization of chemicals at work (ILO, 1993) states that chemical or material safety data sheets (MSDS) contain instructions on the potential hazards of substances and how they can be handled, utilized, and stored safely [11]. Although the significance of MSDS is persistently emphasized, several chemical accidents have occurred because of negligence and a poor understanding of chemical properties. Emphasizing the significance of chemical properties, there have not been many research reports. Lennquist (2012) claimed that accurate information about dangerous chemical substances and their actual health risks must be provided for the safety of workers and for personal protection, firefighting, and first-aid measures [12]. Therefore, acquiring information is a significant factor in reducing the risk of chemical accidents. In 2003 and 2004, the U.S. Chemical safety and hazard investigation board (CSB) conducted accident investigations on three large combustible dust explosions and found that it was not even aware of the risk of dust explosion from the materials utilized and the generation of dust in the production process. In the case of this accident, the operator lacked prior awareness of the risk of such an accident because the lack of adequate information on the combustibility of the slurry-type materials (when dried) [13].

Human factors are classified into workplace and operator factors that positively or negatively affect the performance of workers and act as an important cause of accidents related to hazardous chemicals [14,15]. Several studies have addressed the importance of management of human factors. With approximately 650,000 chemical products placed on the market and hundreds of new products launched each year, many workers face potentially hazardous chemical exposure in the workplace [16]. Nicol et al. (2008) confirmed that 30%–100% of the analyzed products contained chemicals not listed on the MSDS, and the MSDS did not contain all chemicals present, including those known to be serious sensitizers or carcinogens [17]. Similarly, 67% of MSDSs published in 2010–2011 were found to provide insufficient information for engineered nanomaterials [18]. In addition, operators, designers, and safety managers should be properly trained to understand the hazards of materials and reduce human failure of workers by modifying processes where necessary [19]. On the other hand, operator factors depend on individual characteristics and vary according to experience, stress, illness, etc. According to the report of the IAEA (1998), the safety of work can be secured through risk assessment, which can avoid many accidents and environmental disasters. Even in chemical plants, omission of risk assessment when working with hazardous substances can increase the likelihood of a major accident [20]. Gould (2006) claimed that two-thirds of all accidents in the chemical industry were related to maintenance and that failure to work permit (PTW) was the single largest cause. As such, work permits play a key role in ensuring a safe working environment and minimizing human error [21]. Kwon et al. (2015) argued that it is necessary to strengthen the process safety management (PSM) system and implement it because serious industrial accidents caused by chemical substances are continuously occurring due to human error, etc. [22].

Although many chemical accidents have been caused by a combination of human and technical errors, CPI accidents due to human error are unavoidable and difficult to control [23]. Jahangiri et al. (2016) explained that human errors include lack of work permits, inadequate analysis of risks, and inadequate training in the use of safety procedures [24]. Chemical accidents in CPI plants due to chemical labeling problems, lack of understanding of chemicals, or both were also classified as human errors [25,26]. Richardson (1992) claimed that chemical accidents were caused by non-compliance of certain hazardous chemical regulations, and this was caused by ignorance or misunderstanding. Therefore, chemical, physical, and hazardous properties of the product should be well documented and made through proper labeling, documentation, packaging, provision of other precautions, and emergency response procedures [27]. According to previous studies based on the results obtained from analyzing chemical accidents at the sites from January 2008 to June 2018 [28] by the process safety management (PSM), which is in-charge of hazardous chemicals in South Korea, 76.1% of 71 chemical accidents were due to human errors. Chen et al. (2019) investigated human failure mechanisms and derived a model through the analysis of 212 chemical accidents in China. The model confirmed that human errors, such as violations, intellectual limitations, inadequate supervision, and poor safety cultures, were the most essential factors responsible for fire and explosion accidents [29]. Similarly, Zhang et al. (2019) also analyzed that defects in process facilities, which have resulted in accidents and deaths in Chinese oil industries in the past decade, were due to human factors [30].

The purpose of this study was to investigate the correlation between chemical accidents caused by harmful chemicals and human error (e.g., noncompliance or ignorance of regulation) through case analysis of chemical accident investigation reports reported from 2010 to 2017. Improper handling of chemicals, omission of risk assessments and work permits, inadequate training of workers, and misunderstanding of chemicals are commonly classified as human error. Nevertheless, intensive cause analysis and accident case analysis for human failure are required considering the complexity and the impact of chemical accidents. The safety regulations of the UK, a country with a low occupational accident fatality rate, were compared with those of South Korea to confirm the necessary supplements for the system. Four cases of chemical accidents caused by human errors were introduced, and preventive measure to prevent similar accidents were presented. Through these cases, it was shown that a number of accidents involving various types of human errors occur, and preventive measures are required.

## 2. Methods

Among 55 chemical accidents that occurred in South Korea between 2010 and 2017, 41 chemical accidents were classified “human failure related” according to the HSE guideline. It was intended to perform an accurate analysis based on the case of an accident investigation report published in South Korea, which was analyzed using the root cause analysis (RCA) method. RCA methodology is widely used in chemical plants because it helps to identify the root cause of accidents to prevent recurrence of accidental events and to prevent losses and injuries to workers [31]. In addition, representative poisoning, fire, explosion, and leak that occurred in South Korea, which were mixed with various types of human failure, were introduced by analyzing the chemical accident investigation reports.

**Figure 1:**
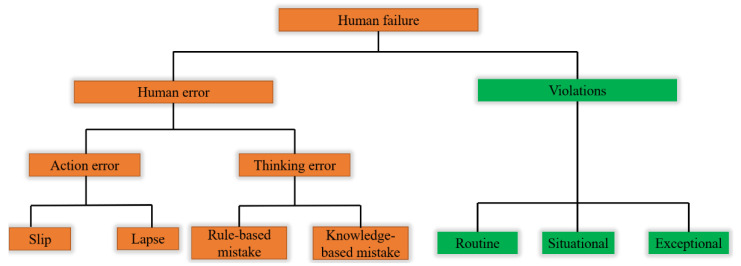
Classification of human failure [32].

According to the UK’s Health and Safety Executive (HSE), human failure can be categorized as human errors and violations as shown in Figure 1 [32]. Human error refers to an unintentional action or decision, while violation refers to intentionally doing something wrong. Violation of safety and health rules or procedures is one of the leading causes of accidents and injuries in the workplace. Human errors can be classified into three types: slip, lapse (skill-based errors), and mistake, and these types of human errors occur in experienced or well-trained persons. Slips and lapses occur in very familiar tasks that can be performed without conscious attention in the workplace, making them very vulnerable to slips and lapses if attention is distracted even for a moment. It commonly occurs during maintenance and repair operations in chemical plants. Slips are called commission errors and are caused by incorrect and often performed physical activities. This happens, for example, in an incorrect operation, such as moving the switch upwards instead of downwards. Lapse is an omission error, and short-term memory loss occurs by omitting procedures or matters necessary to perform essential tasks. Mistakes are decision failures, which are divided into rule-based mistakes and knowledge-based mistakes. These mistakes happen when we do the wrong thing we believe is right and tend to happen when workers do a great deal at once or are running out of time. Rule-based mistakes are made by misapplying good rules or applying bad rules when actions are based on remembered rules and procedures. Knowledge-based mistake refers to the absence of rules or routines that an individual can use to handle unusual situations. For an unfamiliar task, for example, using previous experience. Violation is intentionally doing something wrong and is one of the biggest causes of accidents and injuries in chemical plants by not complying with safety and health rules or process procedures [33]. Routine violations are violations that result from automatic and sometimes unconscious actions. This is a habitual behavior recognized as normal work by a particular workgroup and is often tolerated by organizations and/or governing bodies. Employees may repeat routine violations as they consider their work to be of low risk. Situational violations lead to violations as a result of organizational and environmental factors. These factors include time pressure, lack of supervision, poor ambient conditions (e.g., lighting, noise, heat), lack of resources, and negative culture. Exceptional violations are rare cases that occur in very unusual circumstances, such as emergencies or equipment failures, which result from a conscious decision to violate or an instinctive reaction to the situation.

Among 41 chemical accidents caused by human failure, only 9 chemical accidents were caused by a single cause, but most accidents (about 78%) were caused by multiple causes. If the two cases acted in combination, both were analyzed as the cause. As a result, the number of accidents due to human failure was 41 cases, but the actual number of human failure was 85 cases.

Representative chemical accidents caused by human failure were also shown through case studies. Representative accidents such as poisoning, leakage, explosion, and fire caused by hazardous chemicals were presented in South Korea from 2010 to 2017. Through this accident analysis, the effects of various types of human failure on chemical plants were presented, and the methods of risk management to reduce these chemical accidents were suggested.

## 3. Results

### 3.1. Analysis of Human Errors

**Figure 2:**
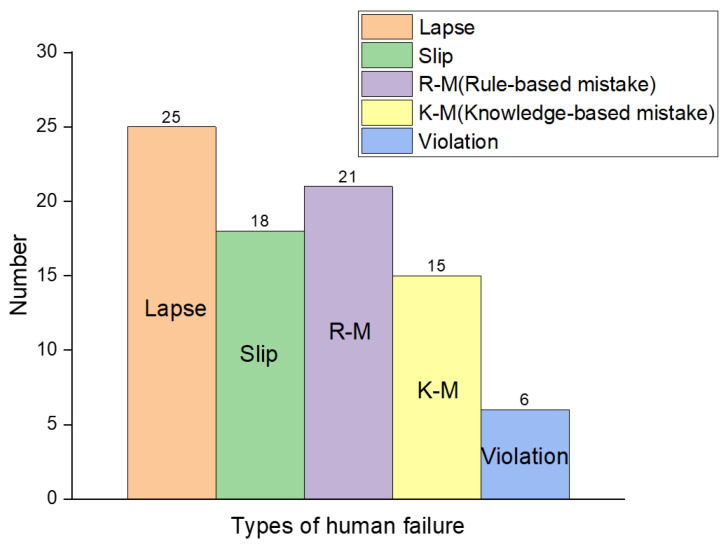
Analysis of human failure in chemical plant during 2010 to 2017.

Figure 2 shows the results of analysis of accidents occurring in chemical plants using RCA analysis. There were only two violations caused by workers’ non-compliance with laws and regulations at the chemical plant, confirming that the safety awareness of workers also improved as the safety law was strengthened. However, as a result of classifying accidents caused by human error, it was found that action errors caused more chemical accidents than thinking errors. It is well known that slips and lapses occurred more frequently with skilled workers, whereas rule-base mistakes and knowledge-based mistakes occurred more frequently with unskilled workers. However, detailed information on the skill level of workers could not be confirmed in the accident investigation report. Lapse in this study was analyzed as one of the most frequent causes of chemical accidents. In many cases, it was analyzed that there was negligence because the risk assessment was omitted to carry out dangerous work, and the work permit was not issued during hazardous work. On the other hand, the eighteen chemical accidents that occurred in chemical plants were caused by slipping, mainly due to work errors of skilled workers. Through the analysis of the accident investigation report, lack of working time, ignoring warnings and alarms that help to detect errors, and workers’ inability to concentrate on their work were the causes of action errors.

It was also found that rule-based mistakes contributed more to chemical accidents than knowledge-based mistakes. This type of human error was often caused by ignoring warnings and continuous work in hazardous situation as well as misapplication of a good rule. The knowledge-based error caused a chemical accident due to incorrect and inappropriate information about chemicals as well as lack of experience in the process. Inaccurate understanding of the exact physicochemical properties of hazardous chemicals was investigated as a factor that caused chemical accidents. In addition, some accidents were analyzed in which workers were not familiar with the process control and were unable to handle abnormal dangerous situations. To prevent this type of accident, preparation of procedures for abnormal and emergency scenarios, knowledge and understanding of chemical processes, and organizational learning are necessary.

**Figure 3:**
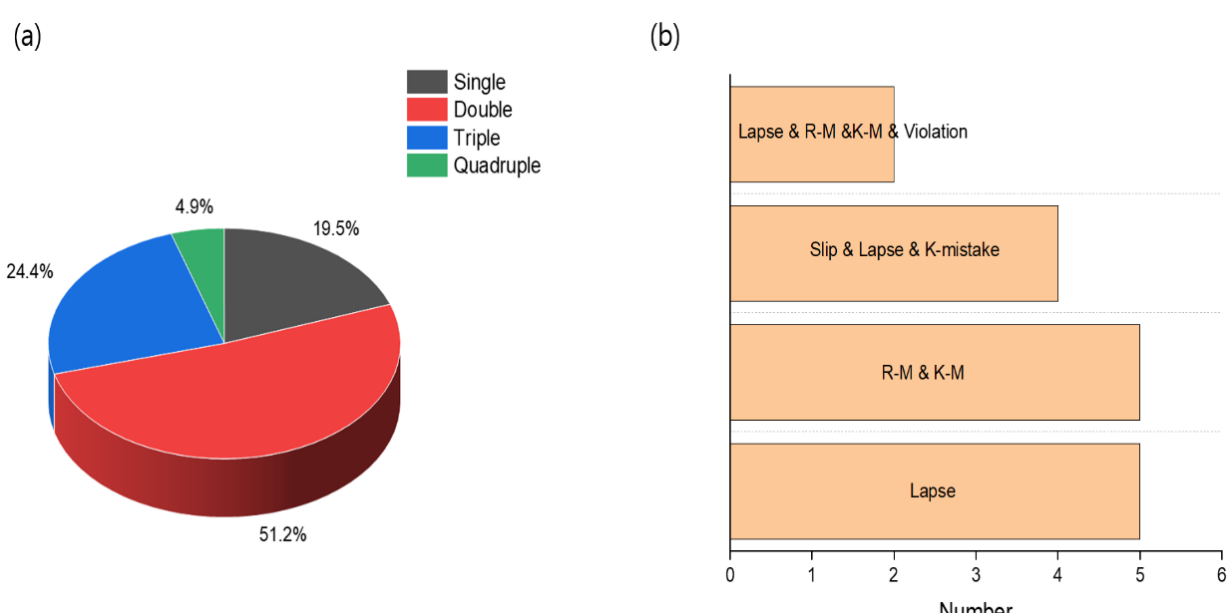
(**a**) Analysis of various factors of human failure and (**b**) analysis of maximum human failure types of single factors and multiple factors.

Figure 3 shows the results of analysis of complex accident types involving more than two human errors out of 41 chemical accidents. According to Figure 3a, 80.5% of accidents occurred due to the complex action of two or more human failures. Among them, accidents involving two causes were the most frequent (51.2%). It was followed by a combination of three human failures, accounting for 24.4%. In Figure 3b, the single cause of lapse-caused accidents occurred the most with 5 cases, and the accidents where the three factors acted in combination were slip, lapse, and knowledge-base, with 4 cases occurring the most. It means that not only are chemical accidents complexly related to equipment and human failures, but also various types of human errors act in a complex way. It means that the prevention of chemical accidents will become even more difficult in the future despite the development of facility safety technology. In order to prevent this problem, it can be seen that significant effort is needed to improve not only the process safety but also the safety culture of workers.

### 3.2. Case Study in Chemical Accident

As the causes of accidents occurring in chemical plants, it is known that equipment failure, human errors, and complex factors interact. Through the chemical accident investigation report from 2010 to 2017 analyzed by the RCA method, cases with various human faiulres were classified by accident type.

#### 3.2.1. Poisoning by Humidifier Disinfectants

Chemical substances such as polyhexamethyleneguanidine phosphate (PHMG-P), chloromethylisothiazolinone/methylisothiazolinone (CMIT/MIT), and oligo(2-(2-ethoxy) ethoxyethyl) guanidinium chloride (PGH) have been employed and commercialized in the production of humidifier disinfectants since 1994, with a total of 14 products sold in South Korea. The products were added to humidifiers (water) and sprayed into the air with the mist. The median legal dose (LD50) of these substances was not high enough to be classified as acutely toxic. These chemicals exhibited no issues if utilized as disposable hand towels. None of the product developers seriously considered the problems and the possible disasters that would result from the lack of a median lethal concentration (LC50) information of the chemical composition of the chemicals, which were employed to spray humidifiers in the air. As discovered after the disaster, LC50s of the chemicals were extremely low and resulted in fatal damage to the respiratory system. According to the latest MSDS from Korea Occupational Safety and Health Agency (KOSHA), updated after the incident, LD50 of PHMG rat oral (858 mg/kg) and rat dermal (2000 mg/kg) as well as LC50 (0.16 mg/l, 4 h, rat) were classified as extremely toxic. The mass of PHMG is 717 g/mol, which is only 5.45 ppm when converted to LC50, and when it is dispersed in air, it exhibits fatal toxicity even under mist, vapor, or gas conditions [34]. Approximately 600,000 humidifier disinfectants were sold each year until an investigation of its possible role in a health hazard commenced in 2011 although the product was not banned during the investigation. Resultantly, lung diseases related to inhalation toxicity increased rapidly from the mid-2000; as of March 2018, there were a total of 6040 victims. Among them, the most recent victims totaled 1421. This was a typical case where the toxicity information in MSDS neither contained any data nor specified a product that should not be commercialized, resulting in public exposure to inhalation toxicity. This tragic disaster occurred because the information required to handle the substance was not accurately documented and utilized in MSDS [30]. According to the special commission on social disaster investigation, there was no inhalation toxicity data in MSDS during the substance review (1997 PHMG, 2003 PGH) by KOSHA, and MSDS was prepared without information about the inhalation toxicity for other humidifier disinfectants, which were sold to residents [35]. From the technical point of view, the main cause of the disaster was the unavailability of the LC50 value of the chemical raw material, which was necessary because of the nature of the product being sprayed into the air; the value was set to “no data”. This value should have been conservatively considered to be “highly toxic” and should have been specified in MSDS for use as “not recommended for use”. However, there was no problem in the regulation because the “no data” item was allowed under the provisions of the law in the inevitable case of no data being available when preparing the MSDS. Currently, the regulations related to this standard have not been modified because of various difficulties, although an improvement in the system is required to prevent similar disasters in the future.

#### 3.2.2. Leakage

**Figure 4:**
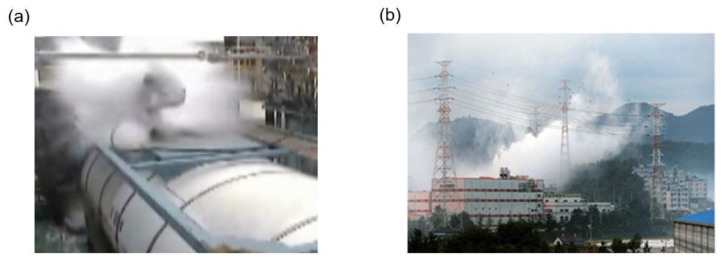
(**a**) Leakage during the operation and (**b**) the spread of anhydrous hydrogen fluoride (HF) around the plant.

In 2012, an HF leakage occurred when a valve was opened while connecting anhydrous HF contained in an International Organization for Standardization (ISO)-graded tank to a chemical plant in South Korea. The leak killed five workers on-site, injured 18 workers in nearby workplaces, and polluted the neighboring environment. Figure 4 shows the leakage scene during the operation and the spread of the gas around the factory. The tragic incident was caused by the leakage of highly toxic substances into the atmosphere with high vapor pressure (1 bar at 19 °C). There were no measures, such as utilization of external structures, connection of scrubbers, emergency protective equipment, and response training, to curb the damages in such cases.

It was stated that the accident investigation report of KOSHA and Gumi city did not contain any information on the risks of chemical substances or the education on the chemical substances handled by workers. Further, the government agencies also failed to promptly evacuate workers and residents from the site or the vicinity after the incident. Rather, they allowed the residents to return to the affected zone even when the concentration of fluoride still exceeded the threshold limit value-ceiling (TLV-C). Consequently, most residents and accident responders (12,000 people) underwent health examinations because of overexposure during the response process. This was because the experts in accident response organizations also lacked the knowledge and the basic understanding of the hazard characteristics of chemicals and the meaning and the application of the level of concern (LOC). If the safety managers and the workers at the workplace had correct understanding of the hazard and the characteristics of chemical substances as well as followed the work procedures, the probability of the accident caused by human error could have been sufficiently lowered. In addition, if the emergency evacuation plan of the accident site had worked properly, secondary damage could have been sufficiently minimized [36].

#### 3.2.3. Explosion

**Figure 5:**
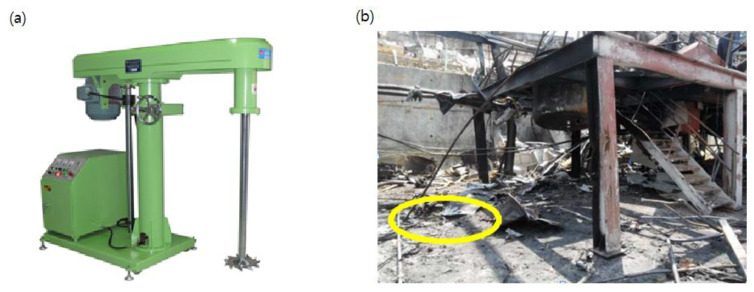
(**a**) Same apparatus as the dissolver used in the event of an accident and (**b**) debris of the burnt factory after the accident. The circled portion in the picture was the position of the dissolver. A small crater from the explosion can still be seen.

A small chemical plant in Gyeonggi-do was testing a mixture of reactive materials to develop a new product in 2012. Unfortunately, a powerful explosion, which resulted in the disappearance of four workers, the injury of nine including the plant manager, and the destruction of the entire factory, occurred. The explosion left a crater, which can still be seen at the site, and the resulting debris spread up to 200 m. The explosion occurred as a result of mixing highly reactive substances, such as benzoyl peroxide (BPO, NFPA reactivity Grade 4), azobisisobutyronitrile (AIBN, NFPA reactivity Grade 3), 2-ethylhexyl acrylate (NFPA reactivity Grade 2), and lithium perchlorate (NFPA reactivity Grade 2) in the dissolver, as shown in Figure 5. According to the accident report by KOSHA, they alleged that anyone could know the name of the substance and search the internet for detailed reaction conditions before proceeding. The unfortunate fact is that the safety managers and the workers at the workplace did not understand the meaning of the National Fire Protection Association (NFPA) responsiveness index. Therefore, they failed to establish risks and protection measures while mixing the chemicals. Through this accident, it can be seen that proper education on the hazards of handling materials and substitution of hazardous processes can reduce the risk of chemical accidents from human error.

#### 3.2.4. Violent Fire

**Figure 6:**
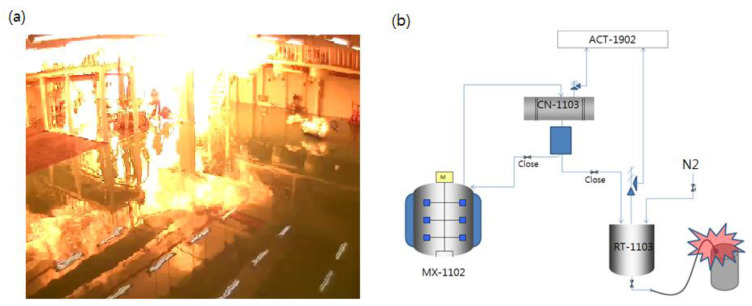
(**a**) Violent fire due to uninformed utilization of 1,4-dioxane and (**b**) schematic of the accident process.

The 1,4-dioxane (NFPA reactivity Grade 1) was utilized as a solvent in the production of the materials for organic light-emitting diodes (OLEDs) in August 2012. A big fire erupted from the 200-L drum, as shown in Figure 6a, while the solvent was being transferred from the recovery tank to the drum, as shown in Figure 6b. The flame was so intense that it swiped the whole processing room. Eight workers died at the site or in the hospital, while three sustained serious injuries from heat and smoke. The flash point (FP) of 1,4-dioxane is 12 °C, which does not generate explosive flames when ignited, as with isopropyl alcohol (IPA), which possesses the same FP. However, the chemical could generate explosive peroxide on exposure to the atmosphere, thereby increasing its concentration and risk when utilized as a solvent in distillation processes, as mentioned in MSDS [37]. In the atmosphere, its ignition energy is significantly lowered, thus increasing its explosive energy and flammability in the presence of an ignition source [38,39]. This finding is in good agreement with the obtained cause of the accident. However, there were clues to the reactivity information in MSDS with an NFPA reactivity rating of one, but even before and after the accident, the company could not retrieve enough additional information. In the accident investigation by KOSHA, it was judged that the reactivity information of the used material was not confirmed, and the risk assessment for the work was omitted. Through accident investigation, it was recommended to conduct a risk assessment in advance for hazardous work that can minimize human error. In addition, improvements such as use of closed systems, monitoring of gas and oxygen in the process, prohibition of grinding work, local ventilation, MSDS education and evaluation, and prohibition of fire sources were recommended. The experimentation of substances without their NFPA reactivity indexes was generally the reality of most workplaces for new product development and is a major limitation to the prevention of explosions in South Korea.

## 4. Discussion

### 4.1. Hierarchy and Causes of Chemical Accidents

According to the scale of general disasters, the ratios of major, minor, and no injury accidents were suggested by Heinrich, and the ratios of deaths, major accidents (MAs), minor accidents, near misses, and unsafe acts were suggested [40,41]. In the case of chemical accidents, the frequency was relatively low, making it difficult to directly apply the ratio with statistics. However, disastrous MAs are an accumulation of small-scale industrial accidents and smaller-scale process accidents. For the root cause analysis (RCA) of these accidents, a flow chart, failure mode and effect analysis (FMEA), Bowtie and Fishbone diagrams, and Pareto chart were introduced along with fault tress analysis (FTA), 5-Whys, and brainstorming techniques for RCA. The KOSHA guideline introduced FTA and RCA techniques, employing the flow chart and the timeline plot for investigations [42].

**Figure 7:**
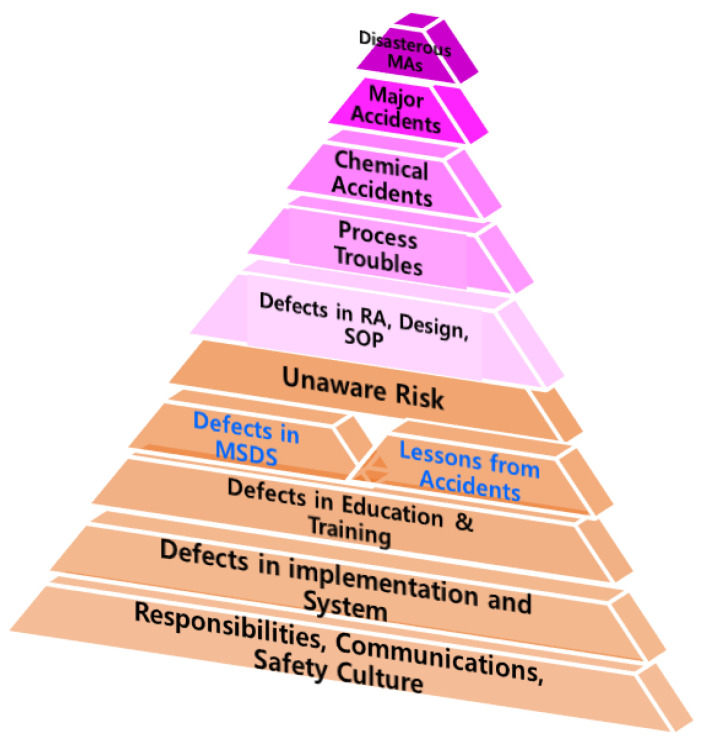
Root cause analysis (RCA) of chemical accidents.

In the analysis of the causes of four accidents, data review, investigation, and analysis of the various processes could be performed. Since the risk characteristics of the chemical itself are related to all risk assessment and prevention, this study was intentionally limited to the contents related to the hazard characteristics of the chemicals. In this case, the main cause of the chemical accident was the worker’s lack of awareness of the hazards of the chemical, and the root cause could be a combination of several human errors, such as not following the lessons of previous similar accidents involving chemicals. The lack or the omission of information on hazardous chemicals to be handled extend to a poor understanding of the information and defects in the preventive measures. Moreover, RCA of these defects is the lack of education and training, which are related to defects in safety and health systems and implementation, which are further attributable to defects in responsibilities, communications, and safety culture. The hierarchical structure of the RCA of chemical accidents analyzed in this study is graphically shown in Figure 7. Unfortunately, the application of the RCA technique to the existing accident investigation report is very limited, thus the use of this technique in the investigation of a chemical accident is essential to derive the exact cause of the accident.

### 4.2. Chemical Hazards and Classifications

Chemical accidents are due to the risks of chemicals, and the risk assessments and the chemical regulations are based on the risks of the chemical substances themselves. The Seveso Directive in Europe, the process safety management (PSM) and the risk management plan (RMP) in the U.S., and the PSM, the RMP, and the safety management system (SMS) in South Korea define the target chemical substances and determine the regulatory quantities in terms of the hazards attributable to each chemical substance [43]. Additionally, the Dow Fire and Explosion Index (F&EI) and the Mond Index, which quantify the risks of chemical processes, were the first to consider material factors [44].

**Figure 8:**
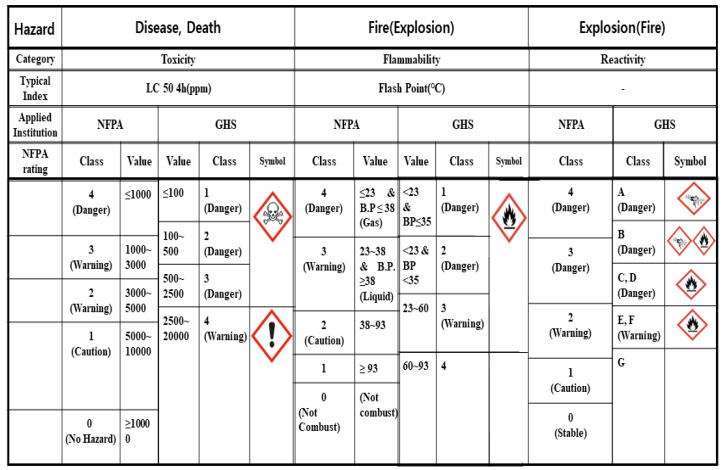
Chemical classification table linking the standard system for the identification of the hazard of materials for emergency response based on the U.S. National fire protection association (NFPA 704) and the globally harmonized system of classification and labeling of chemicals (GHS) standards.

According to the globally harmonized system (GHS) of classification and labeling of chemicals, the risks of chemical substances are classified into 16 groups based on physical risks and 12 groups based on health and environmental hazards [45]. South Korea was also mandated to comply with the GHS system in OSHA since 2010. However, the classification based on the U.S. NFPA 704 (Standard System for the Identification of the Hazards of Materials for Emergency Response) is the simplest, effective, and widely utilized system of classification worldwide [46,47]. It classifies the hazards of a substance into three categories: toxicity (health), flammability (fire), and reactivity (explosion). Furthermore, the levels of these risks are classified as four classes (i.e., 0~4), and most of those for currently utilized chemicals are available in MSDS. Although it was approximated in conjunction with the GHS classification, a classification table (shown in Figure 8) could be drawn. Although the NFPA 704 and the GHS symbols do not completely match, they are easily comparable in risk management. Chemical substances that cause chemical accidents are of class three or higher and are classified as hazardous production materials (HPM). The effectiveness of the HPM classification is adequate. According to the results of the chemical accident investigation, almost all the fire accidents caused by flammable substances were related to classes three or four in flammability category, and poisoning, deaths, or occupational diseases due to chemical substances were denoted as classes three or four in toxicity category. However, it should be noted that, depending on the material and the handling conditions, class one reactive materials could also cause unexpected explosions.

The NFPA classification is very simple and effective in preventing chemical accidents, and its rating for chemicals is mentioned in almost all MSDSs in South Korea. However, according to our investigations and questionnaire results of chemical accidents, only a limited number of workers and safety and health experts understood the actual meanings of the NFPA rating terms and grades, thereby limiting their applications in actual accident prevention and accident cause investigations in particular [48].

The utilization of chemical properties to prevent human error in chemical plants requires the following: (i) the hazards information required for MSDS should be sufficiently and accurately described, (ii) the operator requires basic knowledge to understand and utilize the MSDS information, and (iii) the MSDS information should be utilized when handling chemicals. Government/regulator is responsible for writing, providing, and training workers in MSDS to prevent possible chemical accidents.

Additionally, a PSM system is also required to provide chemical information, such as the chemical abstracts service (CAS) number, exposure limit, explosion limit, FP, ignition point, and vapor pressure. This information is considered to be essential to the risk assessment of the chemical substance. KOSHA conducts professional training on chemical substances for workers, and the Korean government must establish systems that can minimize human error legal regulations. However, statistical investigation is impossible because the proportion of companies that provide training on the risk of chemical reactions with other substances in addition to the training on handling hazardous substances prescribed by law is limited. Taking this into account, it is highly probable that field workers and operators handling hazardous chemicals will not know the NFPA term itself or its exact meaning with regard to chemical risk. This situation acts as a barrier to the prevention of chemical accidents, and there is a risk that future accidents of the same type will recur.

### 4.3. Comparison of Chemical Accident Prevention System between UK and South Korea

To prevent chemical accidents, such as fires, explosions, and leak, it is most basic and important to understand the hazardous characteristics of the chemical substances utilized in major hazardous installation (MHI) and to apply appropriate precautions to prevent human errors. The conclusions of many studies pointed out human errors and equipment malfunctions in the analysis of the causes of chemical accidents and are drawing a lack of understanding of workers’ chemical properties. MSDS provided by the manufacturer of the chemical plays a major role in its understanding, and there is a lack of understanding of the workers who use them and training in the workplace. Therefore, workers must identify and understand precautions in handling hazardous chemicals, and manufacturers must deliver accurate information about physical and chemical properties of hazardous chemicals to prevent major accidents.

South Korea’s PSM, RMP, and SMS regulate the amount of hazardous chemicals handled and stored in the chemical plants. Special chemical facilities with high risk are managed by the government’s safety regulations that stipulate the handling requirements for each chemical substance. The purpose of these legislations should first be the management of chemical hazards followed by the awareness of the hazards attributable to the chemicals to prevent accidents due to handling or utilization of the chemicals in products. In addition, safety procedures and codes of practice for chemical plants are regulated in detail by Korea occupational safety and health agency (KOSHA). Unfortunately, these regulations, safety procedures, and codes of practice are becoming less effective and workable in chemical plants due to the increase of complexities, technological advances, increased maintenance and repair work of equipment, and increased number of dangerous work subcontractors. As of today, efforts to secure the field operability of the PSM and the RMP systems are in progress or have been completed to compensate for these problems.

In most industrialized countries, safety and health approaches are well developed and effective in handling human components in all industries, not just the chemical industry, and safety guidelines and regulations are detailed and mandatory. For example, the UK’s Health and Safety Agency (HSE) is reducing the accident death rate (the ratio of accident deaths per 10,000 people) through the preventive administration and the Health and Safety Act. As of 2019, the fatality rate for all accidents was 0.03 in the UK, 0.37 in the US, 0.14 in Japan, and 0.46 in South Korea. For example, UK safety regulations related to the chemical industry are working effectively to enhance the safety of workers from hazardous substances. The “Health and Safety Information for the Employee Code 1989” aims to raise employee awareness of health, safety, and welfare issues in the workplace, requiring employers to provide their employees with posters or leaflets with information about health, safety, and welfare. The “Management of Health and Safety at Work Regulations 1999” was introduced to strengthen the Health and Safety Act of 1974 and explicitly set what employers must govern in regard to health and safety and apply to all work activities. This regulation imposes a set of obligations on employers and employees to maintain a safe and healthy workplace. The “Control of Substances Hazardous to Health Regulations 2002 (COSHH)” states that it is designed to protect employees and others from the hazards of substances used in the workplace through risk assessment, exposure control, health monitoring, and accident planning.

The occurrence of human errors is related to the understanding of the hazardous chemicals handled. Even if the MSDS provided is complete, it is impossible to prevent chemical accidents unless users and related experts, including regulators, fully understand the contents. If workers handling hazardous chemicals have a good understanding of their hazards, flammability, and toxicity, they can also understand the scale and the impact of potential accidents due to these characteristics. The four case studies discussed here provide examples of how different types of human errors worked together, although their effectiveness was not sufficient to prevent catastrophic chemical accidents.

## 5. Conclusions

In this study, chemical accidents caused by human errors were analyzed from 2010 to 2017, and through four case studies of fire, explosion, leakage, and poisoning caused by domestic hazardous chemicals, it was confirmed that various human errors are complicatedly related. About 80.5% of the accidents we investigated were caused by a mixture of human failures of various types. Human error was found to have a greater impact on chemical accidents than violation, and it was analyzed that more accidents occurred due to action errors than thinking errors. As a result of the analysis of the accident investigation report, workers’ compliance with the laws was well observed due to safety regulations for chemical plants. Nevertheless, it was analyzed that many accidents occurred due to a combination of insufficient understanding and education on the dangers of chemicals, unintentional work mistakes, lack of education, failure to conduct risk assessment, omission of work permits, defects in MSDS, and problems in work procedures. Although many safety measures are installed in chemical plants along with technological development, it is judged that it requires a great deal of effort to prevent accidents in which various types of human failure are combined. In addition, these chemical accidents due to human failure occurred because the field operability of the safety regulations applied to chemical plants in South Korea did not work effectively. If safety regulations are strengthened, and filed management and operability are established as in the UK to prevent such accidents, it will be of great help in preventing chemical accidents caused by human failure. Through case studies of actual chemical accidents in which these complex human failures acted, it is necessary to prepare fundamental measures to not only prevent equipment failures but also human failures, thus preventing chemical accidents.

## Data Availability

Not applicable.
